# Role of lncRNAs in the pathogenic mechanism of human decreased ovarian reserve

**DOI:** 10.3389/fgene.2023.1056061

**Published:** 2023-02-08

**Authors:** Zhexi Lv, Zekai Lv, Linjiang Song, Qinxiu Zhang, Shaomi Zhu

**Affiliations:** ^1^ School of Medical and Life Sciences/Affiliated Reproductive and Women-Children Hospital, Chengdu University of Traditional Chinese Medicine, Chengdu, Sichuan, China; ^2^ State Key Laboratory of Biotherapy, Sichuan University, Chengdu, China

**Keywords:** decreased ovarian reserve, long non-coding RNA, granulosa cell, ovarian function, ovarian dysfunction

## Abstract

Decreased ovarian reserve (DOR) is defined as a decrease in the quality and quantity of oocytes, which reduces ovarian endocrine function and female fertility. The impaired follicular development and accelerated follicle atresia lead to a decrease in the number of follicles, while the decline of oocyte quality is related to the disorder of DNA damage-repair, oxidative stress, and the dysfunction of mitochondria. Although the mechanism of DOR is still unclear, recent studies have found that long non-coding RNA (lncRNA) as a group of functional RNA molecules participate in the regulation of ovarian function, especially in the differentiation, proliferation and apoptosis of granulosa cells in the ovary. LncRNAs participate in the occurrence of DOR by affecting follicular development and atresia, the synthesis and secretion of ovarian hormones. This review summarizes current research on lncRNAs associated with DOR and reveals the potential underlying mechanisms. The present study suggests that lncRNAs could be considered as prognostic markers and treatment targets for DOR.

## 1 Introduction

DOR is defined as a decrease in the quality and quantity of oocytes. The prevalence of DOR in is approximately 10%–35% in women of reproductive age (Liu et al., 2016a). Furthermore, clinical data show that the incidence of DOR is on the rise, and it tends to be younger (Shareghi-Oskoue et al., 2021). The adverse effects of DOR on fertility are becoming more and more serious with the delay of reproductive age. In recent years, decreased fertility caused by DOR has attracted widespread attention. Research on the pathogenesis of ovarian hypofunction is the key to improving female fertility. The etiology of ovarian insufficiency involves its own factors and the influence of the external environment, such as maternal inheritance, unhealthy lifestyles, harmful environmental factors, infection of the pelvic cavity and reproductive system, and surgical injury.

At present, the research on pathogenesis of DOR mainly focuses on the exon region, and little attention was paid to the non-coding regions that constitute more than 97% of the human genome (Shareghi-Oskoue et al., 2021). In the human genome, about 93% of the DNA sequences can be transcribed into RNA, but less than 2% of the nucleic acid sequences are used to encode proteins, and the rest of the transcripts are non-coding RNAs that do not encode proteins. Among multiple non-coding RNAs, long non-coding RNAs (lncRNAs) with a length of 200–100,000 nucleotides are discovered in recent years with the rapid development of the next-generation genome sequencing technology. LncRNAs were founded to play an important role in the pathogenesis of DOR through regulating follicle development and atresia, synthesis and secretion of local ovarian hormones, and affecting the number of follicles and oocyte quality. Therefore, in recent years, lncRNAs have become one of the hot spots in reproductive medicine (Komorowska, 2016). The literature on the role of lncRNAs as key regulators of ovarian dysfunction has rapidly expanded. However, the definite functions of lncRNAs in the pathogenesis of ovarian dysfunction are still unclear and need to be further explored. To facilitate the detection of potential diagnostic markers and therapeutic targets for DOR in clinical practice, enhanced understanding of the role of lncRNAs regulatory network in ovarian function is critical.

Recent studies have shown that the occurrence of ovarian hypofunction is associated with granulosa cell (GC) apoptosis (Gao et al., 2014; Luo et al., 2017; Yin et al., 2018). Granular cell apoptosis can lead to follicular atresia (Shen et al., 2017). Therefore, preventing granulosa cell apoptosis may improve female reproductive lifespan and fertility in patients with DOR. Follicular atresia caused by granulosa cell dysfunction has been widely studied as the main cause of ovarian hypofunction, however, the regulatory roles of lncRNAs in this process have not been systematically sorted out. Therefore, the purpose of this review is to provide an updated narrative review of the roles of lncRNAs in the pathogenesis of female ovarian insufficiency.

## 2 LncRNAs promoting granulosa cell apoptosis

LncRNA PVT1 is a ubiquitously expressed lncRNA that regulates cell cycle, apoptosis, proliferation and migration. Studies have shown that downregulation of PVT1 induces apoptosis of granulosa cells. The mechanism is down regulation of PVT1 enhanced the upregulation of apoptosis-related proteins including Bim, Fas, and FasL, as well as the levels of cleaved caspase-3 and cleaved caspase-8. Forkhead box class O3A (Foxo3a) is a transcription factor belonging to the forkhead family that acts as a trigger of apoptosis in a variety of diseases (Aldonza et al., 2017; Wang et al., 2021). Phosphatase SCP4 is a type of transcription factor that promotes dephosphorylation of Foxo3a and increases its transcriptional activity nuclear phosphatase. In granulosa cells, PVT1, SCP4 and Foxo3a form complexes to exert regulation of apoptosis. Down-regulation of PVT1 inhibits Foxo3a phosphorylation and degradation by promoting SCP4-mediated Foxo3a dephosphorylation. Meanwhile, si-PVT1 could significantly inhibit the degradation of Foxo3a protein, which increases stability and transcriptional activity of Foxo3a, promoting granulosa cell apoptosis (Zhou et al., 2019). It is now considered more likely that PVT1 affects Foxo3a phosphorylation and ultimately regulates its transcriptional activity as the main molecular mechanism of granulosa cell apoptosis. Other studies demonstrated that PVT1 gene could also promote cell proliferation and inhibit ovarian granulosa cell apoptosis by regulating miR-175p and PTEN (Liu et al., 2020).

Studies (Liu et al., 2016b; Zhao and Dong, 2018) have shown that, lncRNA HOTAIR is an accurate and effective biomarker for premature ovarian failure. The expression of HOTAIR in ovarian tissue and serum of patients with premature ovarian failure can accurately predict the risk of premature ovarian failure. However, compared with the expression level in ovarian tissue, serum level of HOTAIR is more accurate. The mechanism by which HOTAIR affects ovarian function is that HOTAIR upregulates the expression of IGF1 by competitively binding to miR-130a, thereby aggravating endocrine disorders and granulosa cell apoptosis. At the same time, HOTAIR may also interact with the Notch pathway which plays an important role in premature ovarian failure. In a mouse model of DOR, activation of the Notch-1 signaling pathway can alleviate DOR-related symptoms, and Notch-1 downregulation has been shown to inhibit granulosa cells growth and promote apoptosis (Lee et al., 2016).

LncRNA DLEU1 was overexpressed in GCs in patients with DOR and acted as a miR-146b-5p sponge to promote granulosa cell apoptosis (Kamalidehghan et al., 2020). DLEU1 and miR-146b-5p can form a base pair and form a direct interaction relationship, which was limited to the cytoplasm. One of the functions of lncRNA is sponge miRNA to alter epigenetics. DLEU1 can spongy miR-146b-5p in the cytoplasm, reducing its apoptosis-suppressing effect, thereby promoting granulosa cell apoptosis. Therefore, overexpression of DLEU1 in GCs may promote disease progression by increasing apoptosis in patients with DOR. It was speculated that DLEU1 and miR-146b-5p may serve as potential targets for the treatment of DOR.

Zhang et al. (2022) found that the expression level of lncRNA TRERNA1 decreased in patients with DOR, and the overexpression of TRERNA1 reduced the apoptosis of KGN cells. Previous studies have demonstrated that miR-23a promoted granulosa cell apoptosis by targeting SMAD5 (Nie et al., 2015), and TRERNA1 and miR-23a formed a potential base-pairing direct binding. Since TRERNA1 overexpression decreased cell apoptosis, while miR-23a overexpression increased cell apoptosis, it was predicted that although TRERNA1 and miR-23a were not mutually regulated, TRERNA1 might inhibit the role of miR-23a in enhancing cell apoptosis. Therefore, TRERNA1 may be involved in DOR by inhibiting granulosa cell apoptosis, and its overexpression may be a potential target.

GC apoptosis is also regulated by many other lncRNAs, such as lncRNA NORFA (Li et al., 2018; Du et al., 2020). LncRNA NORFA inhibits granulosa cell apoptosis by regulating the miR-126/transforming growth factor-β (TGF-β) axis (Zhou et al., 2017). NORFA has been reported to acted as a sponge, that directly bound to the miR-126 to regulate ovarian granulosa cell apoptosis. LncRNA PCAT1 and lncRNA PCAT6 can relieve the inhibitory effect of miR-543 accumulation on cell proliferation, by acting as competitive endogenous RNA (ceRNA) to spongy miR-543 to regulate the expression of SERPINI1 and ZEB1, respectively (Ma et al., 2020; Qu et al., 2020).

The mechanism of action of the above-mentioned lncRNAs is to promote granulosa cell apoptosis by regulating mi-RNA, thereby changing the downstream pathway and other ways, leading to DOR.

## 3 LncRNAs associated with protein translation

In the human genome, lncRNA GCAT1 resides on chromosome 11p11.2 as part of an intergenic fragment between the genes CHST1 and SLC35C1. Studies (Kiyokawa et al., 1996; Rajareddy et al., 2007; Li et al., 2020a) have shown that GCAT1 regulates p27 translation in GCs through competitive binding with PTBP1. Down-regulation of GCAT1 inhibited G1 cell cycle progression, thereby inhibiting the proliferation of GCs. p27 was reported to be an effector of GCAT1 regulating GC proliferation. As a member of the cyclin-dependent kinase inhibitor family, p27 is a well-known inhibitor of cell cycle progression, and reduction of p27 levels in GCs promotes their proliferation and subsequent follicular development and ovulation. GCAT1 initiates the translation of protein p27 by competing with cyclin-dependent kinase inhibitor 1B (CDKN1B) mRNA for binding to polypyrimidine tract-binding protein 1 (PTBP1). Silencing GCAT1 will promote the binding of PTBP1 to the IRES of CDKN1B mRNA, increase the level of p27 in GCs, inhibit cell cycle progression and proliferation of GCs, and lead to follicular atresia and ovarian insufficiency (Cho et al., 2005).

p53 is closely related to DOR as a key player in the regulation of apoptosis, and lncRNA NEAT1 has been shown to be a transcriptional target of p53 (Liu et al., 2015; Idogawa et al., 2017). Li et al. (2020b) found that p53 expression was up regulated in ovarian tissue from patients with DOR compared with that from in patients with normal ovarian function. P53 mRNA expression was negatively correlated with NEAT1 expression. Cells overexpressing NEAT1 showed significantly down-regulated mRNA and protein levels of p53, while cells in which NEAT1 was silenced showed significant upregulation of p53 mRNA and protein levels. In addition, lncRNA-MEG3 could promote proliferation of mouse ovarian granulosa cells which were inhibited by cyclophosphamide (CTX), through regulating the transcription of p53 involved in p66Shc (Levine et al., 2011). Overexpression of NEAT1 may suppress p53 expression and improve ovarian function, and the interaction of NEAT1 with p53 may be therapeutically relevant in patients with DOR (Li et al., 2020b). Therefore, NEAT1 may be a promising diagnostic and therapeutic targets of DOR.

The mechanism of action of the above lncRNAs is to regulate the translation of proteins directly or indirectly in granulosa cells, thereby inhibiting the proliferation of granulosa cells, resulting in the occurrence of DOR.

## 4 LncRNAs associated with gene transcription

LncRNAs have been demonstrated to affect mRNA transcription and protein synthesis. To gain insight into gene activation in granulosa cells, we focused on the mechanism by which lncRNA HCP5, lncRNA-sc1 and lncRNA-sc2 participate in granulosa cell proliferation by regulating the transcription of other genes.

Studies (Mandon-Pepin et al., 2008; Wang et al., 2020) found that the expression of lncRNA HCP5 decreased in GCs of DOR patients, and HCP5 was located adjacent to MSH5 gene. MSH5 is a known causative gene for DOR. HCP5 silencing inhibits the expression of MSH5 and promotes ETO-induced apoptosis of GCs. YB1 protein is a direct binding partner of lncRNA HCP5, additionally, HCP5 stabilizes the interaction between YB1 and its partner ILF2, which can mediate the translocation of YB1 into the nucleus of GCs. HCP5 silencing affects the localization of YB1 in the nucleus and reduces the binding of YB1 to the promoter of MSH5, thereby reducing the expression of MSH5. Combined with previously reported interaction between YB1 and ILF2, it can be concluded that in the nucleus, YB1 normally acts as a transcriptional activator and knocking down HCP5 reduces YB1 binding to MSH5 promoter and inhibits transcriptional activation of MSH5 (Marchesini et al., 2017). Those findings elucidate that HCP5 is regulated by controlling the binding between the YB1 and MSH5 promoters. LncRNA HCP5 transcriptionally regulates MSH5 and DNA damage repair through YB1, resulting in dysfunctional GCs and decreased ovarian function.

Stearoyl-CoA desaturase 2 (Scd2) gene encodes an enzyme that catalyzes the desaturation of fatty acids and is required for lipid synthesis during embryonic skin and liver development. In ovary, saturated free fatty acids negatively affect oocyte maturation and follicle development. Scd2 was reported to expressed at high levels in rat granulosa cells, and it was thought to play an important role in attenuating the toxicity of saturated free fatty acids during oogenesis. LncRNA-sc1 and lncRNA-sc2, both localized in the nucleus of granulosa cells, are transcribed approximately 7 kb and 12 kb upstream of Scd2 gene respectively and promote Scd2 activation directly and indirectly in granulosa cells. However, the mechanism by which these lncRNAs promote Scd2 gene activation is unclear. It is speculated that they may directly interact with Scd2 gene or indirectly participate in regulation of ovarian function (Mayama et al., 2020).

Previous research suggested, after knockout of lncRNA CRYBB2, the ovaries of mice exhibited morphological and functional abnormalities, including decreased ovarian index (ratio of ovarian weight to total body weight), increased follicular atresia, decreased mature follicles, and dysregulated estrous cycles. The expression of cell cycle and apoptosis-related proteins, including B cell lymphoma 2, cyclin-dependent kinase 4, and cyclin D2, is significantly reduced in CRYBB2 knockout mice (Dixit et al., 2006). Another study (Gao et al., 2016) demonstrated that CRYBB2 affected the expression of other lncRNAs, and knockout of CRYBB2 could down-regulate the expression of lncRNA A-30-P01019163, subsequently inhibited the expression of downstream gene P2rx7, and affected ovarian cell signal transduction and cell cycle to change the ovarian structure of mice. However, the precise mechanism by which lncRNA A-30-P01019163 affect P2rx7 translation remains to be resolved.

Loss-of-function mutations in genes responsible for GC proliferation and differentiation, such as FOXL2, 22 BMP15, 23 and WT124, have been found to be associated with DOR. Additionally, lncRNA FoxO3a is often present in the promoters of many pro-apoptotic genes such as Bim and FasL, inducing the transcription of these genes, and then promoting granulosa cell apoptosis (Wang et al., 2012; Zhou et al., 2019).

## 5 LncRNAs affecting cortical tissue of ovary

Lnc-GULP1–2:1 is mainly localized in cytoplasm of granulosa cells. In patients with DOR, the expression of lnc-GULP1–2:1 was low, while in patients with polycystic ovary syndrome it remained a high expression state (Yao et al., 2021a). Lnc-GULP1–2:1regulated KGN cell proliferation by changing the protein expression level and intracellular localization of its target gene collagen III α1 chain (COL3A1), which was a cis-regulatory transcript of lnc-GULP1-2:1. COL3A1 was significantly downregulated in ovarian cortical tissue from DOR patients and could inhibit granulosa cell proliferation by inhibiting cell cycle progression from G1 phase to S phase (Dykes and Emanueli, 2017; Yao et al., 2019). Consistently, downregulation of COL3A1 significantly inhibited granulosa cell proliferation (Gao et al., 2018). The expression and localization of COL3A1 are both regulated by lnc-GULP1–2:1. Studies have shown that lnc-GULP1–2:1 may inhibit cell proliferation by upregulating the expression of COL3A1 and promoting the entry of COL3A1 protein into the nucleus. Lnc-GULP1–2:1 changed the expression level of COL3A1 in GCs and promoted the entry of COL3A1 protein into the nucleus, through this way, affecting the expression level of cell cycle-associated proteins, leading to the inhibition of granulosa cell proliferation.

Xiong et al. (2017) demonstrated that cyclophosphamide could significantly induce ovarian tissue damage, which may be caused by the activation of the p53-p66Shc signaling pathway. Cyclophosphamide can significantly prevent cell cycle progression and inhibit cell division and proliferation *in vitro*. In addition, cyclophosphamide can also induce the activation of p53-p66Shc pathway and increase the expression of apoptosis-related protein (activated caspase-3). In cyclophosphamide-treated mouse ovarian tissue, the expression level of lncRNA-Meg3 significantly increased. It was speculated that cyclophosphamide might induce the activation of p53-p66Shc pathway by activating the expression of lncRNA-Meg3. Moreover, the expression of lncRNA-Meg3 was also shown to increase with the stimulation of cyclophosphamide. It was founded that cyclophosphamide activated the expression of p53-p66Shc pathway-related proteins by inducing the expression of endogenous lncRNA-Meg3 and ultimately possessed an inhibiting effect on granulosa cells, inducing ovarian insufficiency in a mouse model of ovarian dysfunction induced by cyclophosphamide (Xiong et al., 2017).

A study on Hu sheep demonstrated that follicular development-related lncRNA FDNCR, based on the ceRNA (FDNCR-miR-543-3p-mRNA) network, increased decorin (DCN) expression and inhibited transformation growth factor b (TGF-b) signaling pathway, thereby affecting granulosa cell survival and proliferation (Yao et al., 2021b). The study confirmed the mechanism by which FDNCR acted as an upstream regulator of miR-543-3p in GCs to regulate miR-543-3p and prevent the binding of miR-543-3p to DCN 3′UTR, which subsequently released DCN and blocked the TGF-b pathway in order to promote the apoptosis of Hu sheep GC. MiR-543-3p worked as an anti-apoptotic epigenetic regulator in GCs. It regulated GC proliferation through DCN as a direct and functional target. MiR-543-3p-mediated FDNCR increased DCN expression in Hu sheep GCs. DCN inhibited TGF-b by binding to its receptor, thereby interfering with Smad and non-Smad signaling pathway TGF-b receptors downstream of the cell, which suggested that FDNCR regulated GC apoptosis or proliferation by adsorbing miR-543-3p (Comalada et al., 2003; Baghy et al., 2012). Therefore, this study identified a candidate lncRNA (FDNCR) involved in Hu sheep fecundity, providing insight into the regulatory mechanism of follicle development, which would provide the basis for new therapeutic strategies for reproductive diseases.

The above lncRNAs indirectly affect the proliferation of granulosa cells by affecting cells and proteins in the ovarian cortex, resulting in the occurrence of DOR.

## 6 LncRNAs regulating ovarian function through hormones

Anti-Müllerian hormone (AMH) is a glycoprotein closely associated with ovarian dysfunction, and this deficiency results from aberrant regulation of the genes encoding AMH and AMH type II receptor (Amhr2). Specific effects on ovarian function of AMH are exerted *via* its receptor Amhr2. It was found that lncRNA-Amhr2 promoted Amhr2 gene activation in ovarian granulosa cells by enhancing Amhr2 promoter activity. LncRNA-Amhr2 might work only in cis to help contribute to the activation of the Amhr2 gene in mouse ovarian granulosa cells (Kimura et al., 2017). AMH was an important hormone regulating ovarian follicle development, and Amhr2 protein was a key molecule that mediates signal transduction. The findings suggested that lncRNA-Amhr2 was a key molecule controlling AMH signaling through interaction with the Amhr2 promoter in cis and subsequent Amhr2 gene upregulation. The above data support the notion that lncRNA Amhr2 interacts with transcription factors bound to the Amhr2 promoter to activate the gene (Dewailly et al., 2016).

Down-regulated lncRNA ZNF674-AS1 was found in the GCs of DOR patients, which demonstrated that it regulated granulosa cell glycolysis and proliferation by interacting with aldosterone (Li et al., 2021). ZNF674-AS1 acted through interaction with aldolase A (ALDOA), a key enzyme that regulated the process of glycolysis and controls AMP-activated protein kinase (AMPK) in lysosomes activation (Li et al., 2019). The mechanism by which silencing lncRNA ZNF674-AS1 significantly inhibited the proliferation of GCs was as follows: first, silencing of ZNF674-AS1 promoted AMPK activation and regulated the proliferation of GCs through aldolase/v-ATPase-dependent AMPK activation, and second, decreased ZNF674-AS1 expression leaded to decreased aldolase activity and promoted the activation of AMPK, thereby inhibiting the proliferation of GCs. The study revealed a novel interaction between lncRNA ZNF674-AS1 and aldolase and showed that lncRNA ZNF674-AS1 was essential for aldolase activity and glycolytic process in GCs.

lncRNAMALAT1 affected GCs apoptosis and estradiol synthesis through the miR-205/CREB1 axis. Knockdown of MALAT1 significantly increased the expression of miR-205 in GCs, whereas overexpression or downregulation of MALAT1 did not alter MALAT1 expression in GCs. Therefore, it was speculated that MALAT1 might function as a ceRNA by adsorbing miR-205 in GCs, and that overexpression of miR-205 promoted apoptosis and reduced estradiol synthesis in GCs (Zhang et al., 2018; Sun et al., 2021).

## 7 LncRNAs regulating fragile X-related premature ovarian failure (FXPOI)

In addition to the above-mentioned lncRNAs regulating ovarian function, some studies (Elizur et al., 2016; Elizur et al., 2019; Alvarez-Mora et al., 2022) found additional lncRNAs related to a special type of ovarian hypofunction, FXPOI. FXPOI is defined as ovarian failure before age 40, including the process of ovarian dysfunction, and decreased fertility. FMR1 premutation raised the risk for FXPOI in females. LncRNA FMR4, FMR5 and FMR6, which were derived from the FMR1 gene locus, might partake to aspects of the clinical presentation of the FXPOI. Elizur et al. (2016) found that FMR6 displayed a significant non-linear association with the number of CGG repeats in FMR1 premutation carriers (*p* = 0.03). In addition, gene expression level of FMR6 in granulosa cells possessed a negative effect on the number of oocytes in patients with DOR. The research suggested that lncRNA FMR4 produced a toxic gain of function as one of the possible pathophysiologic mechanisms underlying FXPOI. Consistently, Alvarez-Mora et al. (2022) and Elizur et al. (2019) found high expression level of FMR4 was detected in patients with FXPOI, and FMR4 could distinguish FMR1 premutation carrier with FXPOI with a diagnostic power of 0.67. These findings suggested a potential role of FMR4 as a possible biomarker for FXPOI.

## 8 Summary

**FIGURE 1:**
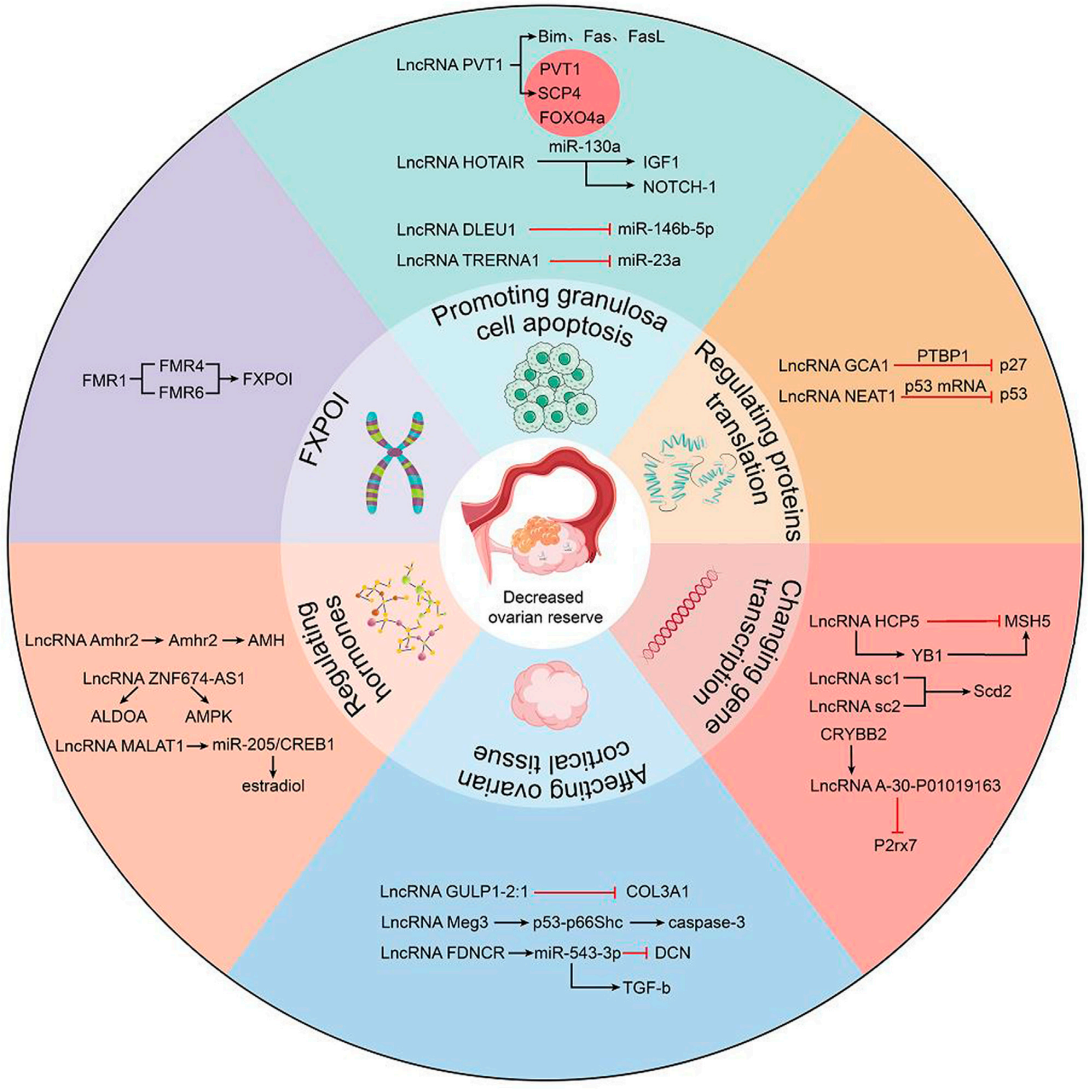
Role of lncRNAs in the pathogenic mechanism of human DOR. LncRNAs may affect ovarian function through the following mechanisms: forming lncRNA-ceRNA networks to regulate transcription of upstream or downstream genes and protein transcription, regulating hormones related to ovarian function by signaling pathways, affecting cortical tissue of ovary, and lncRNAs associated with FXPOI.

LncRNAs as mentioned in the present study are founded to aberrantly express in patients with DOR, such patients displayed clinically subfertility, lower clinical pregnancy rates, higher miscarriage rates and lower live birth rates during assisted reproductive technology treatment. At present, the mechanism of DOR is not yet known, and the regulatory mechanism of lncRNAs in ovarian aging needs to be further studied. Recently, it has been widely accepted that the occurrence of ovarian hypofunction is related to granulosa cell apoptosis. However, the various regulatory mechanisms of lncRNAs in regulating granulosa cell apoptosis have not been systematically sorted out. LncRNAs have emerged as important roles in DOR pathogenesis through diversified mechanisms. Binding with protein, RNA, DNA and other biological molecules, lncRNAs could affect the gene expression of GCs by changing chromatin structure, RNA maturation and transport, protein synthesis and gene expression pathway. The present study specifically discusses the mechanisms by which lncRNAs affect ovarian hypofunction, which are roughly divided into six categories (Figure 1): 1) By regulating signaling pathways or mi-RNAs, such as lncRNA PVT1 (affecting Foxo3a phosphorylation), lncRNA HOTAIR (controls Notch-1 signaling pathway), lncRNA DLEU1 (regulates miR-146b-5p) and lncRNA TRERNA1 (regulates miR-23a). 2) By regulating the translation of proteins in GCs, such as lncRNA GCAT1 (by elevating p27 levels) and lncRNA NEAT1 (overexpressing p53). 3) By enhancing gene transcriptional regulation, such as lncRNA HCP5, affecting the expression of adjacent MSH5 genes. lncRNA sc1 and lncRNA sc2 promoted transcription of the downstream Scd2 gene and lncRNA A-30-P01019163 inhibited downstream P2rx7 expression. 4) Indirectly affect granulosa cells by affecting ovarian cortical tissue, such as lncRNA GULP1-2:1 regulates the expression and intracellular localization of COL3A1, and lncRNA Meg3 induces ovarian tissue damage by activating the p53-p66Shc pathway. lncRNA FDNCR increases DCN expression and inhibits TGF-b transformation. 5) Regulate ovarian function by regulating hormones, such as lncRNA-Amhr2 promotes AMH secretion by activating Amhr2 gene, lncRNA ZNF674-AS1 interacts with aldosterone to regulate granulosa cell glycolysis and proliferation, lncRNA MALAT1 affects mGC apoptosis through miR-205/CREB1 axis death and estradiol synthesis. 6) Special types of ovarian dysfunction, abnormal expression of lncRNA FMR4, lncRNA FMR5 and lncRNA FMR lead to fragile X-related premature ovarian failure. These dysregulated lncRNAs affect the normal function of the ovary through one or several specific mechanisms in GCs.

The discovery of these lncRNAs may provide a new idea for diagnostic and therapeutic strategy to address ovarian dysfunction. However, such studies are immature, so more large-scale cohort trials in different populations are needed to further determine the mechanism of action of lncRNAs. In the present study, it was found that although some lncRNAs displayed abnormal levels of expression in patients with DOR, they have not accurately given the definite value in treatment of DOR. Additionally, the accuracy of lncRNAs in the clinical diagnosis of DOR has not reached consensus.

With the development of RNA sequencing (RNA-seq) techniques, more lncRNAs associated with DOR will be discovered. LncRNAs may become a new indicator for evaluating ovarian reserve function, and its analogs or inhibitors may be used to regulate ovarian aging signaling pathways and intervene in reproductive aging. Basic research achievements in lncRNAs promote its transformation to clinical application and lncRNAs may become a new tool in treatment of patients with DOR.
